# Contribution to the Determination of In Vivo Mechanical Characteristics of Human Skin by Indentation Test

**DOI:** 10.1155/2013/814025

**Published:** 2013-11-13

**Authors:** Marie-Angèle Abellan, Hassan Zahouani, Jean-Michel Bergheau

**Affiliations:** Université de Lyon, ENISE LTDS UMR 5513 CNRS, 58 rue Jean Parot, 42023 Saint-Étienne, France

## Abstract

This paper proposes a triphasic model of intact skin in vivo based on a general phenomenological thermohydromechanical and physicochemical (THMPC) approach of heterogeneous media. The skin is seen here as a deforming stratified medium composed of four layers and made out of different fluid-saturated materials which contain also an ionic component. All the layers are treated as linear, isotropic materials described by their own behaviour law. The numerical simulations of in vivo indentation test performed on human skin are given. The numerical results correlate reasonably well with the typical observations of indented human skin. The discussion shows the versatility of this approach to obtain a better understanding on the mechanical behaviour of human skin layers separately.

## 1. Introduction

Human skin is the largest organ of the human body. The skin protects the body against external influences by preventing fluid loss when exposed to sun, the penetration of undesirable substances in case of pollution, and the development of diseases due to the direct application of external chemical or mechanical loads linked to clinical problems, surgery, or aesthetic treatments. The answers of this barrier to these chemical, biological, mechanical, and thermal loads depend on the person, the site on the body, the age, the health, its nutritional status, its properties, its state (intact or damaged), and its evolutions [1]. Numerous studies have shown that human skin has a stratified structure consisting from the skin outer surface inward of three main layers: the epidermis (composed of the stratum corneum and the viable epidermis), the dermis, and the hypodermis [2–6]. Dryness, microcracks, and loss of elasticity are thought to be influenced by fluid flow and the associated changes in ion concentration as a direct result of mechanical stress states. However, these phenomena are complex to understand and to model due to the strong couplings that exist between them and due to the complex behaviour of the different layers of skin soft tissues. 

Studies of the mechanical behaviour of human skin have observed that the skin is a stratified nonhomogeneous, anisotropic, nonlinear viscoelastic material which is subjected to a prestress in vivo [7–9]. In addition its properties vary with age, throughout the body and per person. Difficulties arise when trying to obtain quantitative descriptions of mechanical properties of the skin. Numerous mechanical experiments have been performed on the skin: tensile testing, suction methods, torsion tests, and indentation experiments [10–16]. For in vivo performed mechanical experiments, the measured behaviour is generally ascribed to the dermis due to the relative height of the viable epidermis and of the stratum corneum. However, the various skin layers are tied together, and it is hard to isolate the contribution of each of them. In vitro experiments give the opportunity to separate the skin layers. But in vitro experimental procedures change the mechanical properties of the individual skin layers. Moreover, it is hard to compare results obtained with different measurement conditions. This illustrates the need for an experimental system to measure the mechanical behaviour of the different skin layers in a noninvasive and objective manner and also independently of the experimental setup. The LTDS-indentation device is able to give a better understanding of the mechanical behaviour of the skin by characterizing the mechanical behaviour of several distinct skin layers in vivo without disturbing its natural stress state before the experiment [15]. 

To achieve this, several experimental setups are developed to load the skin mechanically, theoretical models are derived to describe the experiments, and numerical models are implemented to characterize the mechanical behaviour of the skin layers [17–25]. 

Within the framework of a general phenomenological thermohydromechanical and physicochemical (THMPC) approach of heterogeneous media [22], a triphasic skin model is proposed in [18, 26] which incorporates a solid phase with three solid materials, a fluid phase and an ionic component under ambient constant conditions. Although not negligible, electrical effects are not taken into account in this model. The driving forces for transport are the gradients of the chemical potentials of the fluid and of the ions coupled with the gradients of the displacements of the different solids. In this model, skin is considered as a stratified material with three layers modelling the three outer layers of skin: the stratum corneum, the viable epidermis, and the dermis. All layers of the skin model are supposed to be made of fluid-saturated materials. Furthermore, each layer is seen as a different solid material within the solid phase, and it is described by its own behaviour law. In [18, 26], the solid materials are seen as isotropic linear elastic materials, each of them with its own elastic coefficients. In [27] the solid materials are modelled as nonlinear isotropic Mooney-Rivlin materials with one material constant, leading to the determination of three material constants in total. In [19] the solid materials are described as nonlinear isotropic Mooney-Rivlin materials with two material constants being able to face large deformations. Each solid material has its own strain energy density function of Mooney-Rivlin leading to the need to identify six material constants in total and which allow analysing the decoupled behaviour of each skin layer. These analyses have shown the capability of this model to describe the transient water flow and ion transport through damaged or undamaged skin after application of a saline solution and to gain insight on the mechanical behaviour of human skin layers separately. 

The ionic component models the chemical load applied at the skin outer surface. 

This paper proposes to extend this theoretical-experimental-numerical setup of human skin to the numerical simulation of in vivo indentation test. Skin is seen here as a triphasic medium with four solid materials in the solid phase, a fluid phase, and an ionic component. Furthermore, skin is considered as a stratified material with four layers modeling the four layers of skin: the stratum corneum, the viable epidermis, the dermis, and the hypodermis. All the layers are supposed to be made of fluid-saturated materials and are treated as isotropic linear materials described by their own behaviour law. The governing partial differential equations that arise from the equilibrium, the kinematic, and the constitutive equations are solved under varying physically admissible initial and boundary conditions for the ion concentrations, the fluid, and the solids proposed for describing in vivo indentation test available in the laboratory. A finite difference analysis is carried out which provides a quantitative understanding of water and ion movements through undamaged skin. The numerical simulation allows quantifying the in vivo mechanical properties of the different skin layers of soft tissue separately. 

We will first present the context of this paper linked to the histology of human skin in vivo. Then, to provide a proper setting, we will recapitulate the governing equations for a deforming porous medium including ions. The noninvasive experimental device is presented. Then, example calculations for a specimen of in vivo undamaged skin are given, and the obtained numerical results are discussed. Finally, some concluding remarks are made.

## 2. Histology of Skin

**Figure 1:**
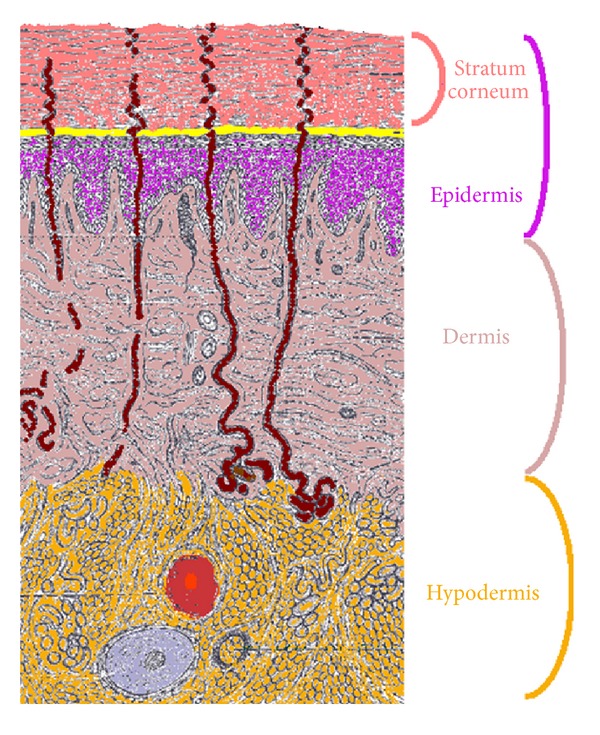
Schematic view of the cross-section of human skin showing the distinct layers.

From the skin surface inwards, skin is composed of stratum corneum, viable epidermis, dermis, and hypodermis (Figure 1). A detailed look to these different layers shows up the following points which are of particular interest in our study. 

### 2.1. Hypodermis

The hypodermis is an adipose tissue composed of loose fatty connective tissue found between the dermis and the muscles. It acts as an insulating layer and a protective cushion. Its thickness varies, some mm, with anatomical site, age, sex, race, and health of the individual. 

### 2.2. Dermis

The dermis can be from 1 to 4 mm thick. It is largely composed of a very dense fibre network of collagen, elastin and minute quantities of reticulin, and a supporting matrix of amorphous ground substance, all bathed in physiological fluid. Physiological fluid provides the cells with nutrients and consists of a water solvent containing mineral and organic solutes as well as waste products from the cells. The dermis contains also microstructures like blood vessels, lymph vessels, nerve endings, sweat glands, sebaceous glands, and hair follicles. The amorphous ground substance combines with the water of the physiological fluid to form a gel which does not leak out the dermis even under high pressure. 

### 2.3. Viable Epidermis

The viable epidermis is a thin (10–100 *μ*m) stratified squamous epithelial of soft keratinized living cells with nuclei, migrating to the outer skin surface: the keratinocytes. The viable epidermis is a nonvascular structure. Cells are surrounded, nourished, and bathed by a physiological fluid originating in the dermis and transported across the epidermal-dermal junction. 

### 2.4. Stratum Corneum

The stratum corneum is a 10–25 *μ*m thick dense coating of hard keratinized dead hexagonal flat cells: the corneocytes, held together by lipid bridges and corneosomes in what is commonly referred to as a brick-and-mortar structure. The corneocytes are the keratinocytes that were migrating to the skin outer surface and that have lost their nuclei. Although the corneocytes are nonviable cells, the stratum corneum is considered to be fully functional, particularly in terms of barrier properties, and retains metabolic functions. Because of its structure and composition, the cells of the stratum corneum have less capacity to bind water than the living cells of the viable epidermis or of the dermis. 

The stratum corneum and the viable epidermis are continuously renewed by desquamation within 6 to 30 days. Cells are shed from the outside and replaced by new ones. 

### 2.5. Consequence on the Skin Model

**Figure 2:**
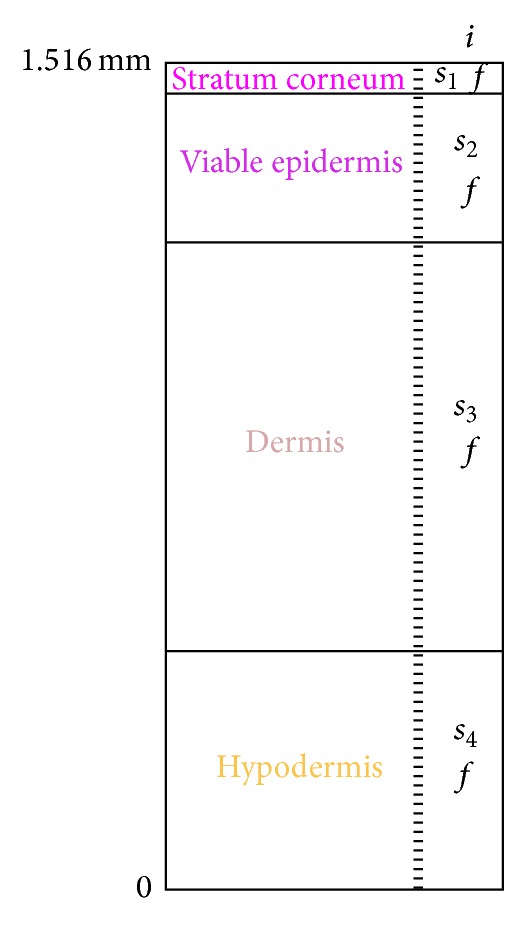
Schematic view of the cross-section of the skin specimen showing the distinct layers and components.

Skin's histology has shown that skin soft tissues are heterogeneous materials consisting of several components. If we combine this statement with the commonly admitted hypothesis that skin is a nonlinear anisotropic hyperelastic and viscoelastic material, the choice is made here to derive the theoretical model for human skin soft tissues seen as a stratified triphasic material with four layers, four solids in the solid phase, one fluid in the fluid phase, and an ionic component.  Figure 2 displays the studied specimen of skin where it is considered that

- the four layers are as follows: layer 1 simulates the stratum corneum, layer 2 for the viable epidermis, layer 3 for the dermis, and layer 4 for the hypodermis,
- the four solids for the solid phase are as follows: solid 1 (*s*_1_) simulates the corneocites and the lipid mortar present in the stratum corneum, solid 2 (*s*_2_) simulates the evolving cells of the viable epidermis, solid 3 (*s*_3_) simulates the different cells of the dermis including the lymph and blood vessels, and solid 4 (*s*_4_) simulates the fatty connective tissue of the hypodermis,
- the fluid (*f*) in the fluid phase simulates the 10% bound water in the lipid mortar of the stratum corneum plus the physiological fluid in the viable epidermis plus the physiological fluid in the dermis plus the physiological fluid in the hypodermis,
- the ionic component (*i*) simulates some cream deposited at the outer surface of the skin either for aesthetic or medical purposes and of which it is relevant to follow the penetration.

The study presented in the following paragraphs is based on this skin specimen. 

## 3. Theoretical Model

Under the phenomenological hypothesis [28], the general THMPC approach of heterogeneous media [22] describes the overall material behaviour of the heterogeneous medium as a combination of the behaviour of each individual component. It is based on the principle of interaction of the components with the following assumptions:

- in each infinitesimal volume of a heterogeneous medium a finite number of components are present;
- each component contributes to the total material behaviour in the same proportion as its volumetric participation given by its volumic ratio;
- all the components are extended to the total studied unit volume of heterogeneous medium.

This approach is applied here to model the human skin specimen introduced in Section 2.5 (Figure 2). 

As it was said, skin is seen here as a triphasic material with four solids for the solid phase (*π*  = *s*_1_, *s*_2_, *s*_3_, *s*_4_), one for each layer, a fluid (*π* = *f*), and an ionic component (*π* = *i*), subjected to the restriction of small displacement gradients, no mass transfer, and no chemical reactions between the constituents; the electrical effects are not taken into account, and the components will be considered as intrinsically incompressible. The studied processes occur isothermally. In addition, the initial configuration of the skin solid skeleton is chosen here as a reference domain for deriving the field equations. Hereafter, all physical quantities are assumed to be functions of the Euler variables (**x**, *t*) where *t* is the time and *x* is the spatial vector defining the position of the material particle in the current configuration at time *t*. Hence, the implicit arguments of all vectors or tensors or scalars are the Euler variables (**x**, *t*). With these assumptions, the source of mass of constituent *π* coming from the other constituents present in the medium is equal to zero, and the balance of mass for each constituent *π* (for *π* = *s*_1_, *s*_2_, *s*_3_, *s*_4_, *f*, *i*) reads

(1) $$\begin{array}{c} \frac{\partial }{\partial t}\rho_{\pi }+\nabla \cdot \left[\rho_{\pi }v_{\pi }\right]=0 \end{array}$$

with *v*_*π*_(**x**, *t*) being the absolute velocity of constituent *π* in m·s^−1^ and *ρ*_*π*_(**x**, *t*) being the relative mass density of constituent *π* in kg·m^−3^ defined for *π* = *s*_1_, *s*_2_, *s*_3_, *s*_4_, *f*, *i* by

(2) $$\begin{array}{c} \rho_{\pi }=n_{\pi }\rho_{\pi }^{\prime }\;, \end{array}$$

where *ρ*_*π*_′(**x**, *t*) is the absolute mass density of constituent *π* in kg·m^−3^ and *n*_*π*_(**x**, *t*)  is the volumic ratio of constituent *π*. As in the remainder of this paper, the subscripts *s*_1_, *s*_2_, *s*_3_, *s*_4_, *f,* and *i* denote the solids, the fluid, and the ions, respectively. The different constituents are supposed to be intrinsically incompressible. Therefore, their absolute mass density are kept constant in this paper. Further, the sum of volumic ratio over the constituents present in the medium should equal one:

(3) $$\begin{array}{c} n_{s_{1}}+n_{s_{2}}+n_{s_{3}}+n_{s_{4}}+n_{f}+n_{i}=1. \end{array}$$

Neglecting inertia forces, convective terms, and the gravity acceleration, the balance of linear momentum for each constituent *π* (for *π* = *s*_1_, *s*_2_, *s*_3_, *s*_4_, *f*, *i*) reduces to

(4) $$\begin{array}{c} \nabla \cdot \sigma_{\pi }+p_{\pi }^{\prime }=0 \end{array}$$

with *σ*_*π*_(**x**, *t*) being the Cauchy stress tensor of constituent *π* in Pa and *p*_*π*_′(**x**, *t*) being the source of momentum for constituent *π* coming from the other constituents in kg·m^−2^·s^−2^, which takes into account the possible local drag interactions between the solids, the fluid, and the ions and which satisfies the momentum production constraint:

(5) $$\begin{array}{c} p_{s_{1}}^{\prime }+p_{s_{2}}^{\prime }+p_{s_{3}}^{\prime }+p_{s_{4}}^{\prime }+p_{f}^{\prime }+p_{i}^{\prime }=0. \end{array}$$

Under the assumption of chemically inert fluid and ions and with solid matrix materials, the material state relation expressing the chemical potential of the fluid *μ*_*f*_(**x**, *t*) encompasses the interactions between solids, fluid, and ions. For the fluid, it reads

(6) $$\begin{array}{c} \mu_{f}=p-\Pi +\psi , \end{array}$$

where *p*(**x**, *t*) is the fluid pressure in Pa, *ψ*(**x**, *t*) is the matrix potential accounting for fluid-solid interactions (capillary and adsorptive effects) in Pa, and Π(**x**, *t*) is the osmotic pressure accounting for fluid-ion interactions in Pa. The chemical potential of the fluid is in J·m^−3^. These quantities need to be experimentally determined in order to write a close mathematical problem, but they are difficult to obtain. Reference [23] considered relations between chemical potential and porosity from different references and proposes the following experimental fit:

(7) $$\begin{array}{c} p+\psi =\left(e^{9.7E-5}\;-e^{\left(9.7E-5\right)/n_{f}}\right)\left(9.84E+10\right), \end{array}$$

(8) $$\begin{array}{c} \Pi =2RTc_{i} \end{array}$$

with *R* being the universal gas constant in J · K^−1^ · mol^−1^, *T* being the absolute temperature in K, and *c*_*i*_(**x**, *t*) being the concentration of the ions per unit fluid volume in mol·m^−3^.

For the ionic component, the chemical potential *μ*_*i*_(**x**, *t*) is defined as

(9) $$\begin{array}{c} \mu_{i}=\mu_{i_{0}}+RT\ln\left(c_{i}\right) \end{array}$$

with *μ*_*i*_0__ being the chemical potential of the ions in a reference state in J·m^−3^. Neglecting couplings between velocity and heat flux, fluid flow through a saturated porous medium with an ionic component is expressed by a generalized Darcy's law hereunder defined in terms of the gradient of the chemical potential *μ*_*f*_(**x**, *t*) of the fluid, the gradient of the chemical potential *μ*_*i*_(**x**, *t*)  of the ions, and a second-order permeability tensor (**x**, *t*):

(10) $$\begin{array}{c} n_{f}\left(v_{f}-v_{s}\right)=-K\cdot \left[\nabla \mu_{f}+\frac{n_{i}}{n_{f}}\nabla \mu_{i}\right]. \end{array}$$

In a matrix-vector notation, the permeability tensor appearing in (10) is a position and time dependent function defined as

(11) $$\begin{array}{c} K\left(x,t\right)=\left[\begin{array}{ccc} K_{xx}\left(x,t\right) & K_{xy}\left(x,t\right) & K_{xz}\left(x,t\right)\\ K_{yx}\left(x,t\right) & K_{yy}\left(x,t\right) & K_{yz}\left(x,t\right)\\ K_{zx}\left(x,t\right) & K_{zy}\left(x,t\right) & K_{zz}\left(x,t\right) \end{array}\right], \end{array}$$

where each component *K*_*ij*_(**x**, *t*) for *i*, *j* ∈ {*x*, *y*, *z*} is a function of position and time and it has the unit m^4^ · N^−1^ · s^−1^.

The diffusion of ions through the fluid phase of a porous medium is taken into account through a Fick's law-type relation by means of a second-order diffusion tensor of the ions *D*_*i*_:

(12) $$\begin{array}{c} n_{i}\left(v_{i}-v_{f}\right)=-D_{i}\nabla \mu_{i}. \end{array}$$

In a matrix-vector notation, the diffusion tensor of the ions appearing in (12) is a position and time dependent function defined as

(13) $$\begin{array}{c} D_{i}\left(x,t\right)=\left[\begin{array}{ccc} {D_{i}}_{xx}\left(x,t\right) & {D_{i}}_{xy}\left(x,t\right) & {D_{i}}_{xz}\left(x,t\right)\\ {D_{i}}_{yx}\left(x,t\right) & {D_{i}}_{yy}\left(x,t\right) & {D_{i}}_{yz}\left(x,t\right)\\ {D_{i}}_{zx}\left(x,t\right) & {D_{i}}_{zy}\left(x,t\right) & {D_{i}}_{zz}\left(x,t\right) \end{array}\right], \end{array}$$

where each component *D*_*i*__*jk*_(**x**, *t*) for *j*, *k* ∈ {*x*, *y*, *z*}  is a function of position and time and it has the unit m^2^ · s^−1^.

The stress-strain relations for the solids are elaborated under the classical assumption for heterogeneous media that the Cauchy stress tensor of the total heterogeneous medium is composed of a solid and a fluid part:

(14) $$\begin{array}{c} \sigma =\sigma_{s_{1}}+\sigma_{s_{2}}+\sigma_{s_{3}}+\sigma_{s_{4}}-pI \end{array}$$

with *σ* being the Cauchy stress tensor of the total heterogeneous medium in Pa and *I* being the second-order identity tensor. 

Under the assumption of small displacements and small strains, skin is considered as a linear isotropic elastic material and a Hooke's stress-strain relation is taken for each solid skeleton (*π* = *s*_1_, *s*_2_, *s*_3_, *s*_4_):

(15) $$\begin{array}{c} \sigma_{\pi }=D_{\pi }^{e}\text{:}\;\varepsilon_{\pi }\;\text{for}\;\pi =s_{1},s_{2},s_{3},s_{4}, \end{array}$$

where *D*_*π*_^*e*^(**x**, *t*) is the elasticity tensor of the solid material *π* in Pa and *ε*_*π*_(**x**, *t*) is the strain tensor of solid *π* defined by

(16) $$\begin{array}{c} \varepsilon_{\pi }=\nabla^{s}u_{\pi } \end{array}$$

with *u*_*π*_(**x**, *t*) being the displacement field of solid *π* in m. It should be noticed that the Cauchy stress tensor, the strain tensor, the elasticity tensor, and the displacement field are functions of the space and time variables. The superscript *s* denotes the symmetric part of the gradient operator.

For later use in Section 5, (15) can be rewritten more conveniently in a matrix-vector notation:

(17) $$\begin{array}{c} \varepsilon_{\pi }=E_{\pi }^{}:\sigma_{\pi }\;\text{for}\;\pi =s_{1},s_{2},s_{3},s_{4} \end{array}$$

with

(18) $$\begin{array}{c} E_{\pi }^{}={\left(D_{\pi }^{e}\right)}^{-1}\;\text{for}\;\pi =s_{1},s_{2},s_{3},s_{4}, \end{array}$$

where *E*_*π*_(**x**, *t*)  is the matrix of the elasticity compliances defined as the inverse of the matrix *D*_*π*_^*e*^ of the elasticity coefficients. Each component *E*_*π*_*ij*__(**x**, *t*) for *i*, *j* ∈ {*x*, *y*, *z*} of the elasticity compliance matrix *E*_*π*_(**x**, *t*) is space and time dependent with unit Pa^−1^. The Cauchy stress and the strain in vector notation are given by

(19) $$\begin{array}{c} \sigma_{\pi }\left(x,t\right)=\left[\begin{array}{c} {\sigma_{\pi }}_{xx}\left(x,t\right)\\ {\sigma_{\pi }}_{yy}\left(x,t\right)\\ \begin{array}{c} {\sigma_{\pi }}_{zz}\left(x,t\right)\\ {\sigma_{\pi }}_{xy}\left(x,t\right)\\ \begin{array}{c} \begin{array}{c} {\sigma_{\pi }}_{yz}\left(x,t\right)\\ {\sigma_{\pi }}_{zx}\left(x,t\right) \end{array} \end{array} \end{array} \end{array}\right],\\ \varepsilon_{\pi }\left(x,t\right)=\left[\begin{array}{c} \begin{array}{c} {\varepsilon_{\pi }}_{xx}\left(x,t\right)\\ {\varepsilon_{\pi }}_{yy}\left(x,t\right)\\ {\varepsilon_{\pi }}_{zz}\left(x,t\right) \end{array}\\ {\varepsilon_{\pi }}_{xy}\left(x,t\right)\\ \begin{array}{c} {\varepsilon_{\pi }}_{yz}\left(x,t\right)\\ {\varepsilon_{\pi }}_{zx}\left(x,t\right) \end{array} \end{array}\right] \end{array}$$

with the elasticity compliance matrix

(20) Eπ(x,t)=[Eπxx(x,t)Eπxy(x,t)Eπxz(x,t)Eπyx(x,t)Eπyy(x,t)Eπyz(x,t)Eπzx(x,t)Eπzy(x,t)Eπzz(x,t)]=[  1Eπ    −νπEπ−νπEπ000 −νπEπ1Eπ−νπEπ000 −νπEπ−νπEπ  1Eπ000 0001+νπEπ00 00001+νπEπ0 000001+νπEπ],

where *E*_*π*_ is the Young modulus of the solid material *π* and *ν*_*π*_ is the Poisson's ratio of the solid material *π*.

The field equations, that is, the balance of mass for the fluid, the balance of mass for the ions, and the balance of momentum for the solids, are complemented by the boundary conditions which hold on complementary parts of the boundary in terms of prescribed external traction, prescribed velocity, prescribed outflow of pore fluid and prescribed pressure, prescribed outflow of ions, and prescribed chemical potential, respectively. The initial conditions which specify the displacements *u*_*π*_ for the solid grains *π* = *s*_1_, *s*_2_, *s*_3_, *s*_4_, the velocities *v*_*π*_ for the solid grains *π* = *s*_1_, *s*_2_, *s*_3_, *s*_4_, and the chemical potentials *μ*_*π*_ for the fluid and the ionic component *π* = *f*, *i* at *t* = 0 close the initial value problem.

## 4. Experimental Device

**Figure 3:**
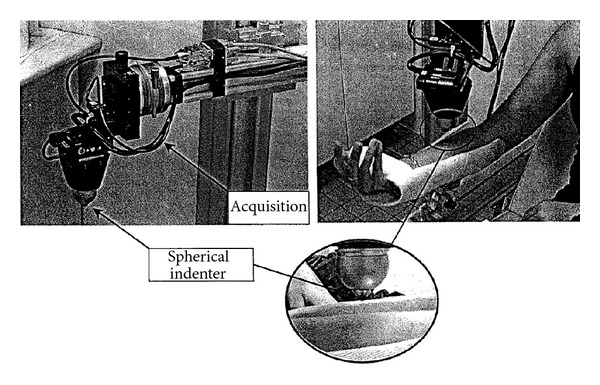
Experimental setup of the LTDS-indentation device.

The original LTDS-indentation device developed by the team of Professor Zahouani permits studying of the mechanical response of the human skin in vivo. This indentation device loads the skin mechanically by applying a controlled normal force onto the surface of the skin. The experimental setup is presented Figure 3. 

**Figure 4:**
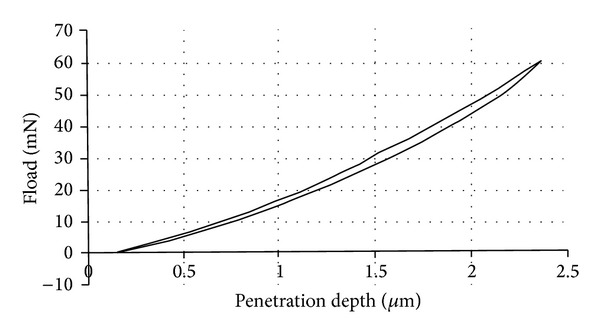
Recorded curve for a loading-unloading indentation test on the volar forearm of an adult.

The penetration depth of the rigid spherical indenter (diameter 6 mm) is recorded as a function of the normal applied force, Fload, during a loading-unloading experiment. The recorded curve for an indentation test performed on the volar forearm zone of a volunteer healthy adult is given in Figure 4. This location is chosen because it is easily accessible, relatively flat, and less disturbed by the natural movement of the body. It makes the indentation tests less tiring for the volunteer because of the position of the arm during the test. Therefore, it disturbs in the least possible way the skin's natural state of stress. The indentation test is realized for a constant indentation speed of 500.0 *μ*m/s at ambient temperature and without surface treatment on skin before the test. 

This loading-unloading curve is reversible with a very low hysteresis due to the dissipated energy. No plastic behaviour is observed, in the sense that there is no residual print onto the surface of the skin allowing the measurement of a plastic depth. Therefore, in this load range, it can be considered that human skin soft tissues can be modeled as elastic materials. 

**Figure 5:**
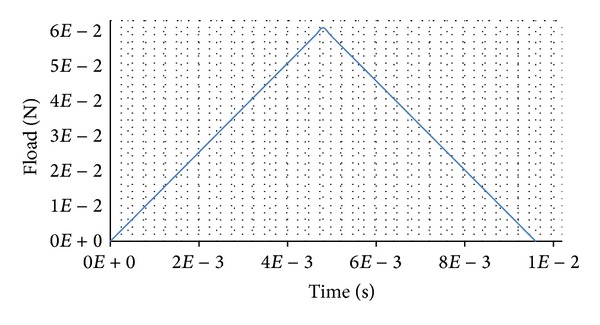
Applied mechanical load Fload versus time for the loading-unloading steps of the numerical simulations.

Moreover, this recorded experimental curve (Figure 4) is reworked and gives a curve of the applied mechanical load Fload versus time for the loading-unloading steps of the numerical simulations (Figure 5).

These experimental data are used in the numerical simulation, hereafter, to define physically admissible boundary and initial conditions and to help characterizing in vivo equivalent mechanical parameters of human soft tissues. 

As it is not possible to measure the thickness of the skin on the inner forearm, we will assume in the following that the thickness of the skin at this part (stratum corneum + viable epidermis + dermis + hypodermis) is approximately 1516 *μ*m (12 *μ*m for the stratum corneum + 102 *μ*m for the viable epidermis + 1002 *μ*m for the dermis + 400 *μ*m for the hypodermis) which are mean values typical for biological soft tissues that can be found already in the literature [29]. Hence, the total response of the skin is the composite response of the individual contributions of the stratum corneum, the viable epidermis, the dermis, and the hypodermis. 

## 5. Numerical Simulation

### 5.1. Finite Difference Model

A finite difference analysis has been carried out that allows for a quantitative understanding of fluid flow and ion transport through intact skin and also of the deformations of the skin layers. The spatial derivatives appearing in the field equations, that is, the balance of momentum (2) for constituents *π*  = *s*_1_, *s*_2_, *s*_3_, *s*_4_, *f*, *i*, the balance of mass (1) for the fluid *π* = *f*, and the balance of mass (1) for the ions *π*  = *i*, are approximated with a second-order accurate finite difference scheme. Explicit forward finite differences are used to approximate the temporal derivatives, which are first-order accurate. As implied in the field equations, the velocities of the solids, the fluid, and the ions are taken as fundamental unknowns and the displacements are obtained by integration when needed.

**Figure 6:**
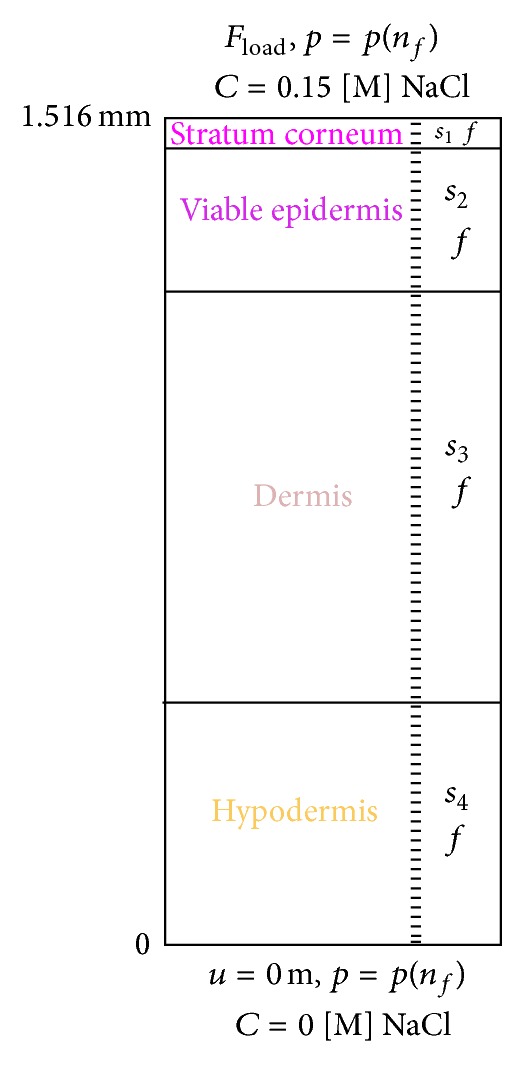
Finite difference mesh and boundary conditions.

The constitutive relations for the solids, the fluid, and the ions are incremental relations giving directly the increment of their associated variable. All calculations are carried out for a specimen of intact skin with a depth of 1516 *μ*m at an ambient temperature of 21°C. The thickness is assumed to be uniform within each layer. The tissue composite consists of 380 elements (Figure 6).

For the simulation, the model needs to be complemented by a set of material parameters. This set is also taken as mean values for human skin available in the literature except as regards of the mechanical parameters for which a mechanical parameters estimation procedure is carried out in Section 5.3. 

### 5.2. Governing Equations

For this 1-Dim numerical simulation of undamaged skin, some equations and relations of Section 3 can be rewritten more simply. They are summarized below with variables that are functions of the vertical Eulerian component *z* of the reference frame.

(i) *Balance of mass for π* = *s*_1_, *s*_2_, *s*_3_, *s*_4_ *, f, i*. Equation (1) reduces to
  (21) $$\begin{array}{c} \frac{\partial }{\partial t}\rho_{\pi }+\frac{\partial }{\partial z}\left[\rho_{\pi }v_{\pi z}\right]=0 \end{array}$$
  with *v*_*πz*_(*z*, *t*) being the vertical component of the absolute velocity of constituent *π*.
(ii) *Balance of momentum for π* = *s*_1_, *s*_2_, *s*_3_, *s*_4_ *, f, i*. Equation (2) can be rewritten as follows:
  (22) $$\begin{array}{c} \frac{\partial }{\partial z}\sigma_{\pi_{zz}}+{p_{\pi }^{\prime }}_{z}=0 \end{array}$$
  with *σ*_*π*_*zz*__(*z*, *t*) being the component of the Cauchy stress tensor of constituent *π* in the *z* direction and *p*_*π*_′_*z*_(*z*, *t*) being the vertical component of the source of momentum for constituent *π*.
(iii) *Generalized Darcy's law for the fluid π* = *f*. Assuming isotropic material, relation (10) leads to
  (23) $$\begin{array}{c} n_{f}\left(v_{f_{z}}-v_{s_{z}}\right)=-K_{zz}\left[\frac{\partial \mu_{f}}{\partial z}+\frac{n_{i}}{n_{f}}\frac{\partial \mu_{i}}{\partial z}\right] \end{array}$$
  with *K*_*zz*_ being the permeability coefficient.
(iv) *Fick's law for the ions π* = *i*. For isotropic material, relation (12) reduces to
  (24) $$\begin{array}{c} n_{i}\left(v_{i_{z}}-v_{f_{z}}\right)=-D_{i_{zz}}\frac{\partial \mu_{i}}{\partial z}, \end{array}$$
  where *D*_*i*__*zz*_ is the diffusion coefficient for the ions.
(v) *Stress-strain relations for the solids π* = *s*_1_, *s*_2_, *s*_3_, *s*_4_. Taking into account relations (19) and (20) into relations (17) and (16) leads to
  (25) σs1zz=Es1εs1zz=Es1∂us1z  ∂z,σs2zz=Es2εs2zz=Es2∂us2z  ∂z,σs3zz=Es3εs3zz=Es3∂us3z  ∂z,σs4zz=Es4εs4zz=Es4∂us4z  ∂z,
  where *σ*_*π*_*zz*__(*z*, *t*) is the component of the Cauchy stress tensor of solid *π* on the *z* direction, *ε*_*π*_*zz*__(*z*, *t*) is the component of the strain tensor of solid *π* on the *z* direction, *u*_*π*_*z*__(*z*, *t*) is the vertical component of the displacement of solid *π*, and *E*_*s*_1__, *E*_*s*_2__, *E*_*s*_3__, and *E*_*s*_4__ are the Young moduli of, respectively, the stratum corneum, the viable epidermis, the dermis, and the hypodermis. The other relations of the theoretical model are used directly without being reworked.

### 5.3. Mechanical Parameter Estimation

**Figure 7:**
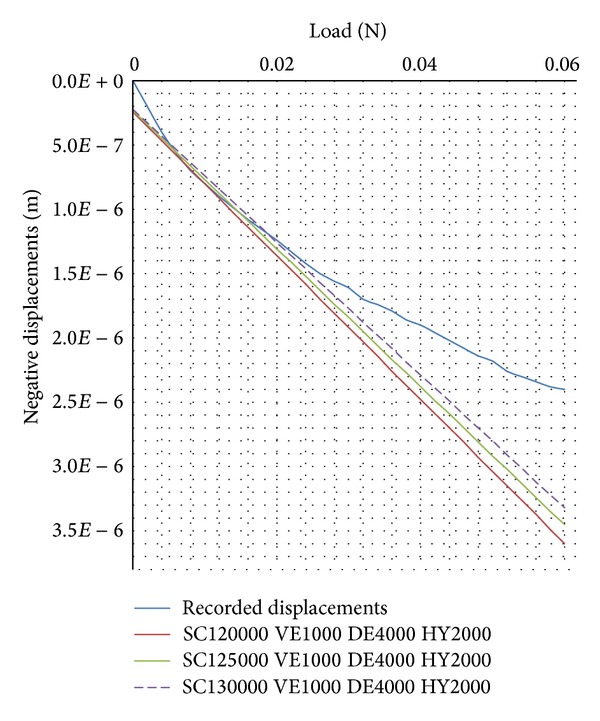
Comparison between numerical displacements curves and the experimental displacement curve for various combinations of material parameters.

An iterative procedure is used to adjust the mechanical parameters of the material model *E*_*s*_1__, *E*_*s*_2__, *E*_*s*_3__, and *E*_*s*_4__ for, respectively, the stratum corneum, the viable epidermis, the dermis, and the hypodermis. Material parameters are set in the input file of the finite difference model. The applied load (Figure 5) is also used as an input in the finite difference model; then, the simulation runs and the numerical results are examined. The computed skin surface displacements during the loading-unloading steps are extracted and compared with the experimentally obtained axial displacement of the skin surface under the indenter.  Figure 7 displays the comparison between the experimental displacement curve and the numerically obtained surface displacements curves for various combinations of discrete values of the material parameters of both layers. The curve “SC120000 VE1000 DE4000 HY2000” corresponds to a simulation carried out with elastic moduli of *E*_*s*_1__ = 120*E*3   Pa for the stratum corneum (SC), *E*_*s*_2__ = 1*E*3  Pa for the viable epidermis (VE), *E*_*s*_3__ = 4*E*3 Pa for the dermis (DE), and *E*_*s*_4__ = 2*E*3   Pa for the hypodermis (HY). The curve “SC125000 VE1000 DE4000 HY2000” corresponds to a simulation carried out with elastic moduli of *E*_*s*_1__ = 125*E*3  Pa for the stratum corneum (SC), *E*_*s*_2__ = 1*E*3  Pa for the viable epidermis (VE), and *E*_*s*_3__ = 4*E*3  Pa for the dermis (DE), and *E*_*s*_4__ = 2*E*3  Pa for the hypodermis (HY). The curve “SC130000 VE1000 DE4000 HY2000” corresponds to a simulation carried out with elastic moduli of *E*_*s*_1__ = 130*E*3  Pa for the stratum corneum (SC), *E*_*s*_2__ = 1*E*3  Pa for the viable epidermis (VE), *E*_*s*_3__ = 4*E*3  Pa for the dermis (DE), and *E*_*s*_4__ = 2*E*3 Pa for the hypodermis (HY).

The comparison shows a minimum difference at approximately the curve “SC130000 VE1000 DE4000 HY2000”.

### 5.4. Numerical Model

Therefore, the skin soft tissues are modelled as isotropic linear elastic materials with an elastic modulus of *E*_*s*_1__ = 130*E*3  Pa for the stratum corneum-simulating solid *s*_1_, *E*_*s*_2__ = 1*E*3  Pa for the viable epidermis-simulating solid *s*_2_, *E*_*s*_3__ = 4*E*3  Pa for the dermis-simulating solid *s*_3_, and *E*_*s*_4__ = 2*E*3  Pa for the hypodermis-simulating solid *s*_4_. An absolute mass density *ρ*_*π*_′ = 1330 kg·m^−3^ is assumed for each solid *π* =*s*_1_, *s*_2_, *s*_3_, *s*_4_. For the fluid, an absolute mass density *ρ*_*f*_′ = 1000 kg·m^−3^ is adopted. For the ions, an absolute mass density *ρ*_*i*_′ = 1549 kg·m^−3^ is taken. In the calculations, the permeability *K* = 1.98*E*−21 m^4^·N^−1^·s^−1^ and the diffusion coefficient *D*_*i*_ = 3.3*E*−11 m^2^·s^−1^ are adopted. 

Environmental conditions are assumed according to the following pattern.

- Upper skin surface: a force is applied at the outer upper skin surface node equivalent to the imposed indentation load obtained from the experimental curve (Figure 5), atmospheric pressure for the fluid, the skin surface is in contact with a 0.15 [M] NaCl solution.
- Inward skin surface: the inward lower surface node is subjected to a displacement condition of 0 mm displacement, a zero flux of fluid, and a zero flux of ions.

Figure 6 gives the initial mesh and the boundary conditions taken in the simulations.

In the initial state, all layers are considered made out of fully saturated material with no ionic component. The calculus starts with an initial state based on experimental data or fit given in [23]. Moreover, with respect to the volumic ratio of the ions, it is set equal to zero for all the layers in the initial state.

## 6. Results and Discussion

For the above set of parameters, the computed numerical results give the evolutions of all the state variables for all the constituents with respect to space and time. They are given and discussed hereafter for some variables in terms of profiles along the specimen of intact skin in vivo. 

**Figure 8:**
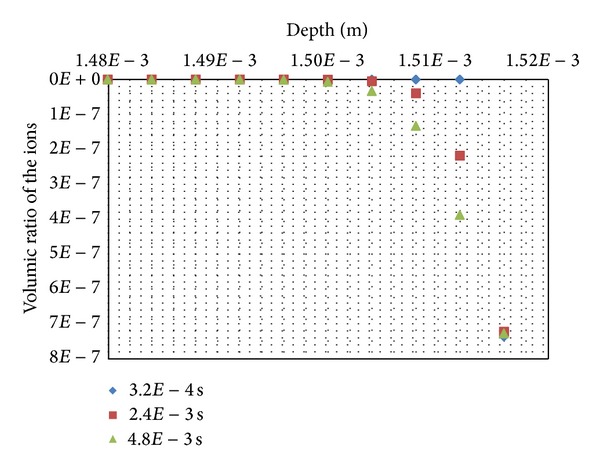
Volumic ratio of the ions.

The volumic ratios of the ions are given in Figure 8 for 3 steps of calculations, *t* = 3.2*E*−4 s, 2.4*E*−3 s, and 4.8*E*−3 s for the upper layers of skin soft tissues. They show a downward movement of the ions penetrating the stratum corneum and also the first layers of the viable epidermis. 

The numerical results show that the ions penetrate till the layer with coordinate 13.92*E*−4 m of the viable epidermis with associated decreasing magnitude of their volumic ratios: 3.69*E*−41 for the layer-simulating node with coordinate 13.92*E*−4 m and 7.27*E*−7 for the upper surface-simulating node with coordinate 15.16*E*−4 m.

**Figure 9:**
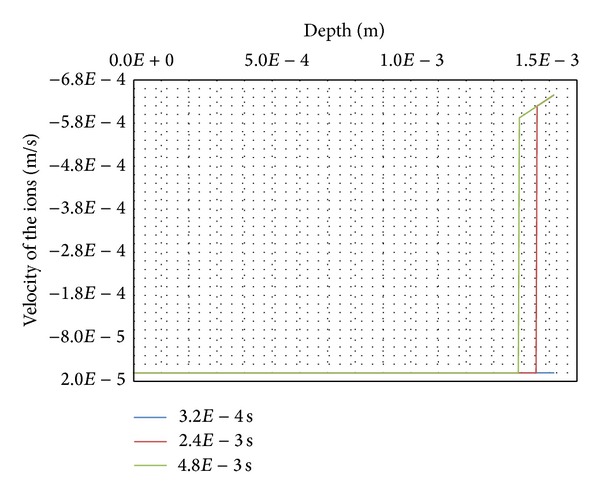
Velocity of the ions.

This penetration occurs with a velocity of the ions shown in Figure 9 for the same 3 steps of calculations *t* = 3.2*E*−4 s, 2.4*E*−3 s, and 4.8*E*−3 s for the upper layers of skin soft tissues. In spite of the low diffusion coefficient of the skin soft tissues *D*_*i*_ = 3.3*E*−11 m^2^·s^−1^, the order of magnitude for the velocities of the ions are quite high −5.92*E*−4 m·s^−1^ for node with coordinate 13.92*E*−4 m and −6.45*E*−4 m·s^−1^ for the upper node with coordinate 15.16*E*−4 m for time 4.8*E*−3 s. Also the velocities tend to increase with respect to time.

**Figure 10:**
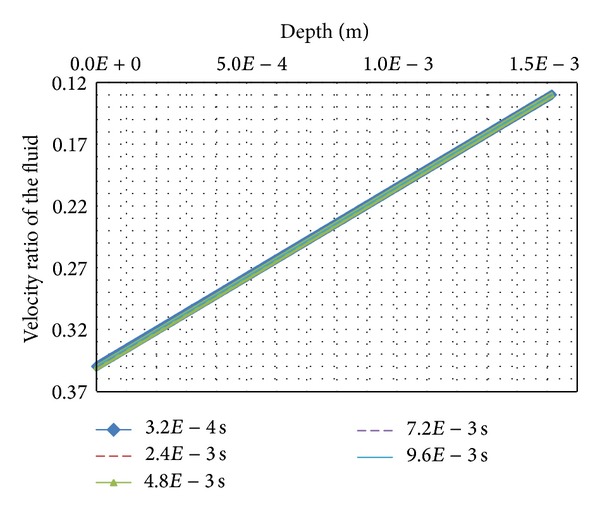
Volumic ratio of the fluid.

Figure 10 displays the linear profiles of the volumic ratio of the fluid for 5 steps of calculations *t* = 3.2*E*−4 s, 2.4*E*−3 s, 4.8*E*−3 s, 7.2*E*−3 s, and 9.6*E*−3 s. The fluid volumic ratio of the skin surface (stratum-corneum-simulating nodes) changes almost immediately after application of the chemical and mechanical external loads. The volumic ratio of deeper layers of the skin (viable-epidermis-simulating nodes and the dermis-simulating nodes) reacts more slowly. The magnitude of these evolutions is low as shown in Figure 10 with the overlapping curves. 

**Figure 11:**
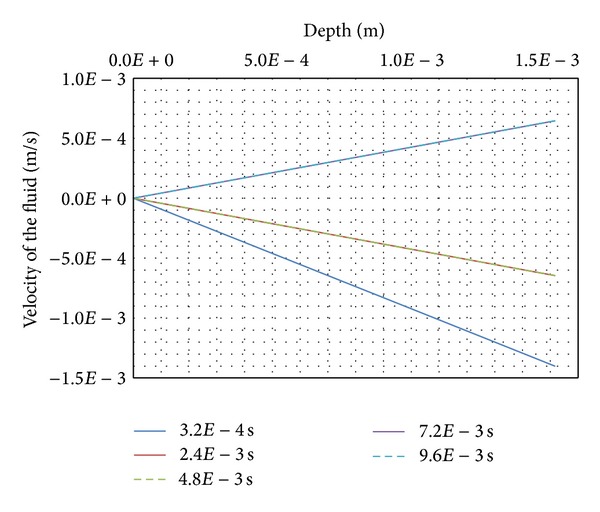
Velocity of the fluid.

Moreover, these evolutions exhibit two different types of movement for the fluid as given by the velocities of the fluid (Figure 11). First the velocity map reveals negative velocities for all nodes of the mesh during the chemical and mechanical loading steps (from 0 s to 4.8*E*−3 s). The fluid is flowing slowly from the upper surface layers inward. Then, the velocities become positive for all nodes of the mesh during the unloading steps (from 4.8*E*−3 s to 9.6*E*−3 s). The fluid tends to flow upward in search of a new equilibrium. 

**Figure 12:**
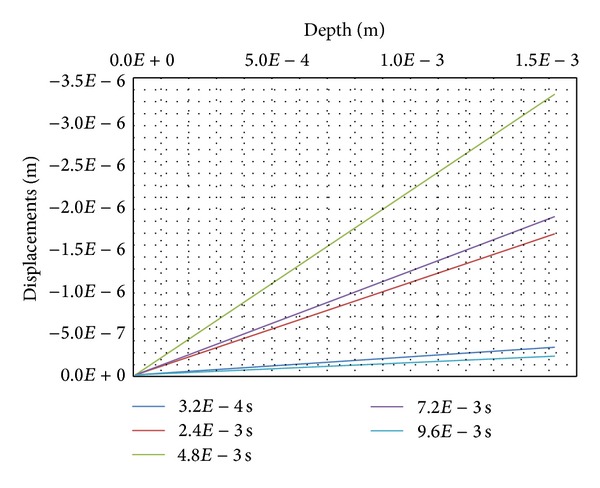
Displacements of the solids.

The negative displacements of the solids are presented in Figure 12 for 5 steps of calculations *t* = 3.2*E*−4 s, 2.4*E*−3 s, 4.8*E*−3 s, 7.2*E*−3 s, and 9.6*E*−3 s. During the chemical and mechanical loading steps (from 0 s to 4.8*E*−3 s), the solids display a consolidation-type behaviour all along the specimen of skin soft tissues-simulating nodes. 

On the contrary, during the unloading steps (from 4.8*E*−3 s to 9.6*E*−3 s), the magnitude of the relative displacements becomes higher and the volume of the specimen increases. In spite of the choice of an isotropic linear elastic behaviour for all the skin tissues-simulating solids, it can be observed some kind of viscosity behaviour because the state at the end of the unloading steps has not yet come back to the initial one (curve 3.2*E*−4 s and curve 9.6*E*−3 s are not overlapping each other). 

The initial state taken as an input for the specimen of human skin is based on experiments. Therefore, it includes physiological influences and couplings which are not yet taken into account in our model but which are impossible to decouple. An example is relations (7) and (8) used for the calculus of the chemical potential of the fluid. Nowadays, it is accepted that fluid pressure is generated by the heart. It pushes water out of the capillaries. On the other hand, water potential is created due to the ability of small solutes to pass through membranes. This movement induces osmosis, that is, water passing from a high concentration (of water) outside to a low concentration inside in an attempt to reach an equilibrium. There, osmotic pressure drives water back inside. Because of the heart, the physiological fluid is constantly flowing and equilibrium is never reached. Because of the differences between fluid pressure and osmotic pressure, the flow of water pushes water outside again and influences the imbalance in solutes, favoring the net movement of physiological fluid. This influence on our model can be seen in Figure 7 at the beginning of the loading steps and at the end of the unloading steps. The studied specimen of skin is not in equilibrium at the beginning of the loading steps. It does not reach an equilibrium at the end of the unloading steps. A zero applied mechanical load Fload does not lead to a zero computed numerical penetration depth because the couplings between solids-fluid-ions are still imposing nonzero fluid and ions internal loads resulting in these nonzero negative displacements.

The applied chemical and mechanical external loads at the upper outer surface of the skin specimen lead to gradients of concentration of ions and to gradients of deformations for the different solids. This leads to nonzero velocities for ions initiating the penetration of the ions in the upper layers of the stratum corneum. It gives also nonzero velocities for the fluid which starts flowing downward from the upper surface of the stratum corneum to the deepest layers of the hypodermis. In search of equilibrium, new values of the fluid pressure influence the magnitude of the chemical potential of the fluid and of the chemical potential of the ions. Through the hypothesis made with relation (13) on the definition of the Cauchy stress tensor of the total medium, the evolutions of each solid is then triggered by these coupled phenomena.

Comparison with results from the literature is not easy in case of nonlinear material behaviour like in vivo skin. However, making an attempt of comparison, it can be said that the numerical results obtained with the analysis lead to qualitatively equivalent evolutions when compared with the typical observation of indented human skin in vivo [20, 21]. The dense coating of hard keratinized dead cells of the stratum corneum is rich in insoluble keratin proteins. It gives the stratum corneum its very strong tensile strength. The stratum corneum transmits almost entirely the received external mechanical load. 

The viable epidermis is a stratified epithelial of soft living cells. It is very souple and answers the transmitted part of the external mechanical load by a consolidation-type behaviour. The viable epidermis takes a big part of this transmitted load.

The dermis is made up of collagen and elastin fibers embedded in a gel. The interwoven collagen fibers provide strength while the rubber-like elastin fibers account for the skin's elastic behaviour. The gel protects the soft living cells from the transmitted external mechanical load till a yield pressure is reached. The coupled behaviour of the solid matrix of the dermis and the gel leads to a consolidation-type behaviour of the dermis with a magnitude lower than the one of the viable epidermis.

The loose fatty cells of the hypodermis reacts almost like a rubber mattress with a reversible behaviour in an answer to the transmitted external mechanical load (as shown in Figure 12 with the overlapping beginning of curve 3.2*E*−4 s and curve 9.6*E*−3 s for the hypodermis-simulating nodes).

## 7. Conclusion

This paper has presented a triphasic skin model where skin is composed of four solids, a fluid, and an ionic component. Despite the large number of simplifications and assumptions, it is shown that this model is able to give information on the transient fluid flow and ion transport through skin layers when the outside surface of the skin is in contact with a saline solution. Based on this model, the numerical simulation performs reasonably well in describing indentation experiment. The estimation procedure, for the Young modulus of each layer, resulted in *E*_*s*_1__ = 130*E*3 Pa for the stratum corneum, *E*_*s*_2__ = 1*E*3 Pa for the viable epidermis, *E*_*s*_3__ = 4*E*3 Pa for the dermis, and *E*_*s*_4__ = 2*E*3 Pa for the hypodermis. To the author's knowledge, the obtained values can be said to be realistic for the stratum corneum, the dermis, and the hypodermis. A separate Young modulus for the viable epidermis has not yet been reported. However, it is thought to be physically reasonable because of the qualitatively good agreement between the obtained numerical descriptions of the overall response of the skin specimen and the experimental observations available in the literature. 

In conclusion, the theoretical and numerical model presented here enable capturing deformations of the different layers of the skin composite separately. It offers perspectives for the in vivo determination of the mechanical properties of skin soft tissues.
